# Correction: Reliable prediction of *n*-heptane isomerization over Pt/(CrO_*x*_/ZrO_2_)-HMS *via* comparative assessment of regularization networks and surface response methodologies

**DOI:** 10.1039/d0ra90081h

**Published:** 2020-08-04

**Authors:** Nastaran Parsafard, Ali Garmroodi Asil, Shohreh Mirzaei

**Affiliations:** Kosar University of Bojnord Iran n-parsafard@kub.ac.ir; University of Bojnord Iran; Ferdowsi University of Mashhad Iran

## Abstract

Correction for ‘Reliable prediction of *n*-heptane isomerization over Pt/(CrO_*x*_/ZrO_2_)-HMS *via* comparative assessment of regularization networks and surface response methodologies’ by Nastaran Parsafard *et al.*, *RSC Adv.*, 2020, **10**, 26034–26051, DOI: 10.1039/D0RA04313C.

The authors regret that the one of the affiliations (affiliation b) was shown incorrectly in the original manuscript. The corrected list of affiliations is as shown here.

The Royal Society of Chemistry apologises for these errors and any consequent inconvenience to authors and readers.

## Supplementary Material

